# Teacher-reported emotional and behavioural problems and ethnic background associated with children’s psychosocial care use: a longitudinal population-based study

**DOI:** 10.1007/s00787-021-01937-w

**Published:** 2022-01-10

**Authors:** D. G. M. Eijgermans, H. Raat, P. W. Jansen, E. Blok, M. H. J. Hillegers, W. Jansen

**Affiliations:** 1 The Generation R Study Group, Erasmus MC, University Medical Centre, Rotterdam, The Netherlands; 2 Department of Public Health, Erasmus MC, University Medical Centre, Rotterdam, The Netherlands; 3 Department of Child and Adolescent Psychiatry/Psychology, Erasmus MC, University Medical Centre, Rotterdam, The Netherlands; 4 Department of Psychology, Education and Child Studies, Erasmus University Rotterdam, Rotterdam, The Netherlands; 5 Department of Social Development, City of Rotterdam, P. O. Box 70032, 3000 LP Rotterdam, The Netherlands

**Keywords:** Psychosocial support systems, Psychosocial intervention, Social determinants of health, Health services needs and demand, School teachers

## Abstract

Approximately, 15% of children in Western countries suffer from emotional and behavioural problems. However, not all children receive the psychosocial care they need, especially children with a non-Western background experience an unmet need for care. This might be because parents of non-Western children report a lower need for care than parents of Western children, unrelated to the actual need. This study examined the association between teacher-reported problems and psychosocial care use, independent of mother-reported problems. Further, the role of ethnic background in this association was investigated. The study sample of 9-year-old children was retrieved from the Generation R Study (*N* = 3084), a prospective, population-based cohort of children born in Rotterdam, the Netherlands. Teacher- and mother-reported problems were measured via questionnaire when the children were  6/7 years old. Psychosocial care use was mother-reported at the research centre when children were 9 years old (8.1%). Hierarchical logistic regressions showed significant positive associations between teacher-reported total, externalising and internalising problems and later psychosocial care use. These associations were independent of mother-reported problems. Children with a non-Western background used less care, but ethnic background did not moderate the association between teacher-reported problems and care use. Our findings suggest that teachers might have an important role, next to parents, in the identification of problems and children’s access to care. This may be particularly important for non-Western children, as they use less psychosocial care than Western children, despite other research showing that they generally display higher levels of problems. Directions for future research and implications are discussed.

**Supplementary Information:**

The online version contains supplementary material available at 10.1007/s00787-021-01937-w.

Approximately, 15% of the children in Western countries suffer from emotional and behavioural problems [1–4]. The prevalence of these problems in Western countries seems to be higher among children with a non-Western background than children with a Western background [5, 6]. It is, therefore, worrying that children with a non-Western background receive, on average, less psychosocial care than their Western peers [7–10]. A possible explanation of the lower psychosocial care use among these children is the perceived need for care. Several studies have shown that, regardless of the mental problems, the perceived need for psychosocial care is lower in non-Western people than in Western people, including children [11, 12].

Apparently, children’s need for care is not always interpreted in the same way by parents, while the recognition of problems by parents is an important step in the pathway to psychosocial care [13]. Hence, other important adults, e.g. teachers, may play an essential role in recognising children’s emotional and behavioural problems [14, 15]. Teachers might have a different perception of the children’s need for psychosocial care than parents, because they view children's emotional development and behaviour in a different setting and in the context of peers. As such, teachers may support children and their families in the pathway to care, which may ultimately help to attenuate the ethnic background differences in children’s psychosocial care use. However, only little research has been performed on the association between teacher-reported emotional and behavioural problems and children’s psychosocial care use. It is important to understand—yet not studied before—whether this association depends on the parent-reported problems, because the differences in care use between ethnic groups are partly explained by the differences in parents’ recognition of problems [11, 12].

Three population-based, longitudinal studies on this subject show contradicting findings. Raven et al. [16] found that teacher-reported internalising problems at 11 years old were associated with increased care use in the age range of 11–13 years, but that teacher-reported internalising problems later in adolescence and externalising problems overall were not associated. Sourander et al. [17] identified the teacher’s reported need for referral at 8 years old as a determinant for psychosocial care use at 16 years old. However, both studies did not investigate the role of ethnic background in the associations. The third study, by Erath et al. [9], did include ethnic background and focussed solely on externalising problems. In this study, teacher-reported externalising problems during kindergarten were associated with increased care use. This study showed that children with a European-American background used more psychosocial care than children with an African-American background. However, no interaction between ethnic background and externalising problems was found.

It has been shown that ethnic background is a moderator in the association between parent-reported problems and psychosocial care use; children were more likely to use care when they had a Western background compared to a non-Western background [9, 18, 19]. However, whether the association between teacher-reported emotional and behavioural problems and psychosocial care use is moderated by ethnic background is unclear since the literature is scarce and inconclusive. This study aims to investigate whether teacher-reported emotional and behavioural problems are associated with children’s psychosocial care use, and whether this association is independent of parent-reported emotional and behavioural problems. Subsequently, this study aims to investigate whether children’s ethnic background moderates the possible association between teacher-reported emotional and behavioural problems and children’s psychosocial care use. This study includes teacher-reported total, externalising and internalising problems. By studying these aims, we will provide insight into the teachers’ possible role in early detection of the need for care in young children, also for children with a non-Western background. Furthermore, this study could contribute to improve prevention strategies and facilitate early intervention [20].

## Methods

### Data collection

We used data from the Generation R Study, a prospective, population-based cohort study of children born in Rotterdam, the Netherlands, which has been described elsewhere [21]. In this cohort, 9749 children and their parents are followed from foetal life onwards. In total, 5862 participants visited the research centre for the measurement wave at 9 years between 2011 and 2014. Children who dropped out of the study were more likely to be boys, have a non-Western background, and have parents with a low educational level compared to the remaining sample [21]. Children with complete data on psychosocial care use and teacher-reported problems were included in the current study. Thus, participants were excluded because of missing data on psychosocial care use (*N *= 363) or teacher reports (*N *= 2415), resulting in a sample of 3084 participants. The Medical Ethics Review Board of the Erasmus Medical Centre approved the study protocols. All parents provided written informed consent.

### Measures

*Psychosocial care.* Parents were asked about psychosocial care use of their child at 9 years old. The question at the research centre was: ‘Has your child in the past twelve months been examined or treated for any mental health problem by a professional?’ Eight different types of psychosocial care were distinguished, i.e. five forms of specialised mental health care, social work, preventive youth health care, and the general practitioner. Visiting only preventive youth health care or the general practitioner was recoded into using no care (16%) because they mainly provide preventive care or refer, but do not offer treatment for emotional and behavioural problems. The remaining six care providers were combined to ‘psychosocial care use in the past year’.

#### Teacher-reported emotional and behavioural problems

The Teacher Report Form (TRF), 6–18 years, was used to measure the children’s emotional and behavioural problems [22]. The TRF was filled out by the teacher of the participants when the children were on average 6.7 years old and consists of 112 items scored on a three-point scale. The TRF shows good validity and reliability [22]. The crude TRF scores were adjusted for residual differences regarding age and sex. Hereafter, the 83rd percentile cutoff points, based on the total Generation R sample, were used to determine the total problem score’s borderline or clinical range. Children scoring on or above this cutoff were classified as having emotional and behavioural problems according to the teacher. Since we have a relatively healthy sample, we will describe this as ‘having problems above the 83rd percentile’ instead of ‘having borderline/clinical problems’. The same procedure was followed for the internalising and externalising problems scale.

#### Ethnic background

Ethnic background of the child was measured via a questionnaire when the children were 6 years old. Children were classified as having Western background when both parents were born in a Western country [23]. If one or both parents were born in a non-Western country, the child was classified as having a non-Western background.

#### Covariates

To test whether the associations were independent of potential confounding factors, we adjusted for sex (boy vs girl), educational level of both parents (high vs mid-high vs mid-low vs low), and family situation (two-parent family vs one-parent family). These sociodemographic factors were also accounted for in the previously described population-based, longitudinal studies on this topic [9, 16, 17]. Additionally, we adjusted for age at the outcome measurement, based on the day of visiting our research centre. Furthermore, to investigate the independent association of teacher-reported problems, we adjusted for mothers’ parent-reported problems. The mother-reported emotional and behavioural problems were obtained via de Child Behavior Checklist (CBCL/1½–5) when the children were on average 6.0 years old. The CBCL consists of 99 items [24]. The same residual and cutoff procedures as for the TRF were followed.

### Analyses

Missing values on the covariates were imputed 20 times using multiple imputation methods. A significance level of *p* < 0.05 was maintained in all analyses. Analyses were performed using IBM SPSS Statistics 25.

Descriptive statistics were used to summarise the characteristics of the study population. The association between teacher-reported emotional and behavioural problems, ethnic background and psychosocial care use at 9 years old was studied using hierarchical logistic regression analyses. The variables were entered as follows: teacher-reported problems and ethnic background were studied in univariate analyses. In the next step, Model 1, teacher-reported emotional and behavioural problems and ethnic background were entered together. Model 2 consists of the Model 1, and age at visit of the research centre (at time of outcome measurement), sex, educational level of mother and father, and family situation. Model 3 consists of Model 2 and mother-reported emotional and behavioural problems. The hierarchical models were applied three times: once with the TRF total problems score, once with the TRF externalising problems score and once with the TRF internalising problems score. To study the possible moderating role of ethnic background, the interaction term between teacher-reported problems and ethnic background was added to Model 3.

#### Additional and complete case analyses

As additional analysis, the moderating role of mother-reported problems was studied by adding the interaction term between teacher- and mother-reported problems to Model 3. The association between teacher-reported problems and psychosocial care use might differ for children whose mothers do and do not report emotional and behavioural problems, as parents most often seek psychosocial care for their children [13]. A complete case analyses was performed to investigate whether the imputation influenced the results.

## Results

**Fig. 1:**
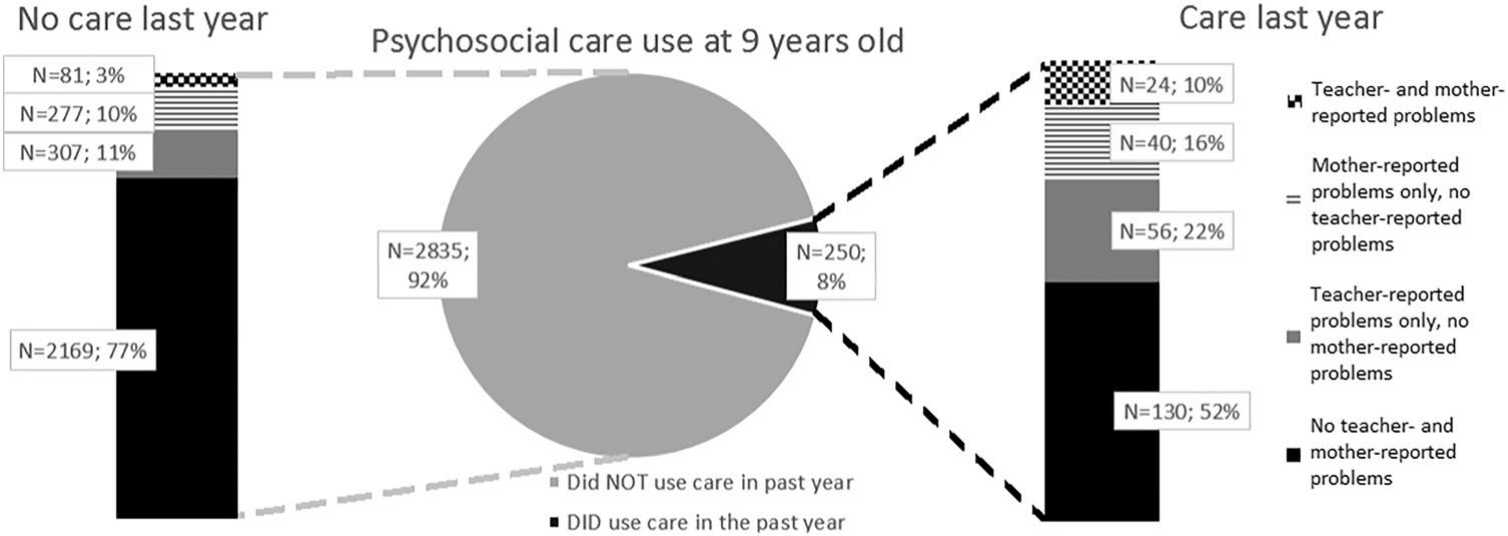
Teacher- and parent-reported problems at 6 years old, stratified for children that do and do not use psychosocial care

Children in the sample were on average 9.8 (SD 0.3) years old, the majority (68.0%) had a Western background, and almost half (49.4%) were boys. More sociodemographic characteristics and the percentages of emotional and behavioural problems above the 83rd percentile are described in Table 1. Psychosocial care was used in the past year by 8.1% of the children. Among those children who used psychosocial care in the past 12 months (age nine years; *N *= 250), 10% had emotional and behavioural problems above the 83rd percentile at 6 years old according to both the teacher and the mother (see Fig. 1). A higher percentage only had problems according to either the mother (16%) or the teacher (22%). More than half of the children that used care (52%) had no problems at 6 years old according to the mother and teacher. A similar pattern was present among the children that did not use care in the past year: at age 6 years, a small percentage had problems above the 83rd percentile according to the teacher as well as the mother (3%), and a larger percentage according to either the mother (10%) or the teacher (11%).

**Table 1:** Characteristics of the total study sample (*N *= 3084)

Data before imputation	*N* (%)	Miss.%	Percent of group that used psychosocial care in the past year
Psychosocial care use in the last 12 months	250 (8.1)	*0.0*	100
Age at visit to research centre (in years)	9.78 (± .33)		N.A
Ethnic background			
Western	1952 (68.0)	*6.9*	9.0
Non-Western	920 (32.0)		6.4
Sex			
Boy	1523 (49.4)	*0.0*	10.1
Girl	1561 (50.6)		6.1
Educational level mother			
Low	423 (14.8)	*7.2*	5.7
Mid-low	895 (31.3)		9.8
Mid-high	767 (26.8)		9.8
High	777 (27.1)		6.3
Educational level father			
Low	443 (17.1)	*16.0*	8.4
Mid-low	698 (26.9)		9.7
Mid-high	608 (23.5)		8.6
High	841 (32.5)		7.1
Family situation			
Two-parent family	2319 (86.8)	*13.3*	8.2
One-parent family	354 (13.2)		8.8
Teacher-reported emotional/behavioural problems above the 83rd percentile			
Total problem score			
Yes	468 (15.2)	*0.0*	17.1
No	2616 (84.8)		6.5
Externalising score			
Yes	476 (15.4)	*0.0*	19.3
No	2608 (84.6)		6.1
Internalising score			
Yes	514 (16.7)	*0.0*	12.5
No	2570 (83.3)		7.2
Mother-reported emotional/behavioural problems above the 83rd percentile			
Total problem score			
Yes	406 (15.4)	*14.3*	15.8
No	2237 (84.6)		7.1

Percentages are valid percentages and, therefore, add up to 100% without the missing values

*Miss.* missingness within the variable

The numbers in Italics represent the percentage of missing values within the variable

### Teacher-reported emotional and behavioural problems and psychosocial care use

The presence of teacher-reported total, externalising and internalising problems above the 83rd percentile were all associated with psychosocial care use in 9-year-old in the univariate analyses (*p* < 0.05, see Table 2). Children with total problems according to the teacher were more likely to receive care when adjusted for sociodemographic factors [odds ratio (OR): 3.06; 95% confidence interval (95% CI): 2.26–4.15]. Children with teacher-reported externalising problems were also more likely to receive care (OR 3.84; 95% CI:2.86–5.17). The association for children with teacher-reported internalising problems showed a lower—but still significant—odds ratio (OR 1.86; 95% CI 1.37–2.53).

**Table 2:** Multivariable hierarchical logistic regression with presence of teacher-reported emotional and behavioural problems at 6 years old, ethnic background and psychosocial care use at 9 years old (*N *= 3084)

	Univariate analyses OR (95% CI)	Model 1 OR (95% CI)	Model 2 OR (95% CI)	Model 3 OR (95% CI)
*Overall problem level*				
Teacher-reported problems				
Presence of total problems^a^	**2.97 (2.23–3.95)**	**3.23 (2.41–4.33)**	**3.06 (2.26–4.15)**	**2.83 (2.09–3.86)**
Ethnic background				
Western (ref.)	1.00	1.00	1.00	1.00
Non-Western	**.69 (.51–.94)**	**.60 (.44–.82)**	**.60 (.43–.85)**	**.58 (.41–.82)**
Mother-reported total problems	**2.41 (1.76–3.31)**	–	–	**2.10 (1.51–2.93)**
*Externalising problems*				
TRF				
Presence of externalising problems^a^	**3.72 (2.81–4.91)**	**3.95 (2.98–5.24)**	**3.84 (2.86–5.17)**	**3.59 (2.66–4.85)**
Ethnic background				
Western (ref.)	1.00	1.00	1.00	1.00
Non-Western	**.69 (.51–.94)**	**.60 (.44–.83)**	**.63 (.44–.88)**	**.61 (.43–.86)**
Mother-reported total problems	**2.41 (1.76–3.31)**	–	–	**2.06 (1.47–2.87)**
*Internalising problems*				
TRF				
Presence of internalising problems^a^	**1.82 (1.35–2.46)**	**1.91 (1.41–2.59)**	**1.86 (1.37–2.53)**	**1.75 (1.28–2.39)**
Ethnic background				
Western (ref.)	1.00	1.00	1.00	1.00
Non-Western	**.69 (.51–.94)**	**.66 (.48–.89)**	**.62 (.44–.87)**	**.60 (.42–.84)**
Mother-reported total problems	**2.41 (1.76–3.31)**	–	–	**2.25 (1.63–3.11)**

Bold: represents *p* ≤ 0.05

*OR* odds ratio for psychosocial care use at age 9 years, *95% CI* 95% confidence interval, *TRF* Teacher Report Form

^a^Teacher-reported total problems above the 83rd percentile. Model 1: ethnic background, TRF total problem score. Model 2: Model 1 + age at visit research centre (outcome), sex, educational level mother and father, family situation. Model 3: Model 2 + mother-reported emotional and behavioural problems

### The association independent of mother-reported problems

Mother-reported problems were added in Model 3. This resulted in attenuated, but still significant, associations between the teacher-reported problems and care use, i.e. for total problems (OR 2.10; 95% CI 1.51–2.93), externalising problems (OR 2.06; 95% CI 1.47–2.87), and internalising problems (OR 2.25; 95% CI 1.63–3.11). Mother-reported total problems above the 83rd percentile were also significantly associated with psychosocial care use, independently of teacher-reported problems (see Model 3 of Table 2).

### Children’s ethnic background in this association

Children with a non-Western background were less likely to use psychosocial care in all models compared to children with a Western background (see Table 2). The association between children’s ethnic background and psychosocial care use remained stable and significant after adjusting for all covariates in Model 3, i.e. having a non-Western background was associated with less care use (e.g. OR 0.58; 95% CI 0.41–0.82 in the analysis with total problems). None of the interaction terms between teacher-reported problems (total, externalising and internalising problems) and ethnic background showed a significant *p* value in Model 3 (all *p* > 0.05, see Supplement SI).

### Additional and complete case analyses

In the additional analyses, we tested whether the association between teacher-reported problems and psychosocial care use was different for children of mothers who do and do not report problems by adding interaction terms. None of the interactions between the teacher-reported total, externalising and internalising problems and mother-reported total problems were significant (all *p* > 0.05, see Supplement Table SI).

The complete case analyses showed that all odds ratios were in the same direction as in Model 3 of Table 2 (see Supplement Table SII). On average, the associations were slightly stronger for the total (OR 3.52; 95% CI 2.50–4.96), externalising (OR 3.84; 95% CI 2.74–5.38) and internalising problem level (OR 2.04; 95% CI 1.45–2.88), compared to the analyses on the imputed data. The association between children’s ethnic background and psychosocial care use remained stable compared to the imputed data, i.e. having a non-Western background in the analysis with total problems (OR 0.58; 95% CI 0.40–0.85), with externalising problems (OR: 0.63; 95% CI 0.43–0.92), and with internalising problems (OR 0.63; 95% CI 0.43–0.92). None of the interactions was significant in the complete case analyses (*p* > 0.05, results not shown).

## Discussion

Our study’s main findings are that teacher-reported total, externalising and internalising problems above the 83rd percentile (i.e. representing borderline/clinical problems) at 6 years old were associated with psychosocial care use at 9 years old. This association remained significant after adjusting for mother-reported total problems and was not moderated by ethnic background of the child. This association was also not significantly different for mothers that did and did not report emotional and behavioural problems of their child as we found no interaction effect between teacher- and mother-reported problems. Further, children with a non-Western background used less psychosocial care compared to children with a Western background, unrelated to their problem level.

Our study findings are in line with previous studies on differences in psychosocial care use between children with a Western and non-Western background [7–10]. To our knowledge, the study of Erath et al. [9] is the only study investigating the potential moderation by ethnic background in the association between teacher-reported problems and care use. They only investigated teacher-reported externalising problems. They, too, did not find a moderating role for ethnic background, while care use was significantly lower among children with a non-Western background. An explanation introduced by Ivert et al. [25] pertains to differences in the pathways to care. They show that the referral to psychiatric clinics in Swedish (non-migrant) children is most often initiated by their parents, while in migrant children with a South American or Asian background, referrals were more likely to be initiated by school or the health-care sector. These different pathways might be explained by acculturation issues that parents with a migrant origin have to deal with, e.g. language barriers and the knowledge of the health care system [26, 27]. Research shows that those experiencing the most issues are the least likely to receive mental health care [26, 28]. These issues differ according to country of origin and decrease by time of residence [28, 29]. These acculturation issues might explain the differences in the pathways to care and why ethnic background does moderate the association between parent-reported problems and mental health care use [9, 18, 19], but not the association between teacher-reported problems and care use.

Other explanations on the relative lower care use by children with a non-Western background are present in the literature as well. Nanninga et al. [30] found that non-Dutch parents prefer to solve the psychosocial problems of their child themselves and that they have less confidence in the treatment and therapists than Dutch parents. Flink et al. [31] described how children with a Turkish and Moroccan background in the Netherlands fear the potential negative reactions from parents and friends regarding their psychosocial care use, including disappointment, worrying, or shame. These negative attitudes towards care and fear of stigma are related to reduced help-seeking activities [32]. Johnson et al. [33] showed that parents with minority backgrounds in the USA experience greater barriers to mental health care, e.g. transportation to the care facility, and also rate the quality of care lower.

Our study findings on the associations of the teacher-reported total, externalising and internalising problems at 6 years old with psychosocial care use at 9 years old are in line with previous studies on this association [9, 17]. The current study adds to the findings of previous studies by its focus on psychosocial care use in childhood, while the other studies focus on adolescence. This might also explain the differences with the study by Raven et al. [16], who only found an association for teacher-reported internalising problems in early adolescence, but not for internalising problems in later adolescence or externalising problems. Additionally, the current study shows that the association of teacher-reported problems and psychosocial care use is independent of mother-reported problems. This implies that the recognition of problems by the teacher can be of added value next to the recognition of problems by parents. To our knowledge, no other studies adjusted for parent-reported problems.

In our study sample, psychosocial care in the past year was received by 8.1% of our 9-year-old sample. This is somewhat lower than the registered care use of 10% in 2015 in the Netherlands [34], which can probably be explained by our relatively healthy study sample. Remarkably, more than half of the children (52%) that received care in the past year did not score above the 83rd percentile on emotional and behavioural problems at 6 years old, according to the teachers and mothers. Only 10% of the children who received care did have a score above the 83rd percentile according to the teacher as well as the mother. Of the children that used care, 22% had only teacher-reported problems and no mother-reported problems. In more detail, for children with a Western background, this percentage was 19%, and for children with a non-Western background, this percentage was 33% (data not shown). This implies that teachers may especially play an important role in identifying emotional and behavioural problems by children with a non-Western background. Additionally, a recent study—on the association between mental disorders and teacher concerns about children’s mental health in British children—also concludes that teachers can play a valuable role in referral decisions as teacher concerns proved to be moderately predictive of disorders [35]. This provides opportunities as currently in the Netherlands teachers formally have no active role in the screening on mental health problems.

The discrepancy between the teacher-reported emotional and behavioural problems at 6 years old and psychosocial care use at 9 years old is considerable. On the one hand, only a small percentage of the children (17%, see Table 1) with teacher-reported problems above the 83rd percentile does receive care. On the other hand, the majority of the children receiving care did not have the highest teacher-reported problem scores (68%, see Fig. 1). It is possible that emotional and behavioural problems in children reported at age 6 diminished or increased between the age of 6 and 9. However, it is not likely that these changes explain the entire discrepancy [36]. Further, children with high problem levels might already have been treated for their problems before the age of 9. Again, this is also not likely to be the full explanation, as only 13.7% of the children with teacher-reported problems did receive earlier care (data not shown). Another explanation why children without high problem levels use care might be that the TRF and CBCL do not fully cover the reasons for psychosocial care use by children. However, the most likely explanation is that, in practice, not all children with the most problems and highest needs receive psychosocial care, while conversely a substantial number of children who receive care may not have the highest needs [37].

### Strengths and limitations

This study has several strengths. First of all, we studied a young sample of children. This age category has not often been studied since most studies so far focussed on adolescents [38]. Secondly, we studied a general population to increase the generalisability of our findings and to be able to inform public health policies better. Furthermore, we used a longitudinal design in a large cohort which strengthens the findings. Using the longitudinal design increases the likelihood that teacher-reported problems were present before the care was received. Lastly, we adjusted for mother-reported problems. Recognition of problems by parents often is the first step in the help-seeking process. Because we adjusted for mother-reported problems, our results show the added value of the teachers’ problem recognition.

This study also has some limitations. First of all, teacher-reported problems might be biased by the rest of the school class [39]. Teacher-reported problems can be influenced positively and negatively by school and class-related factors. Secondly, the teacher- and mother-reported problems are not filled out at the exact same point in time. However, they are both adjusted for age to correct for this. Lastly, our findings should be generalised with caution. The cohort of Generation R had some drop-out over the years, resulting in a relatively healthy sample [21]. Next to the drop-out, we only included children data on teacher-reported problems. The excluded children were more likely to use less care, have a non-Western background, have lower-educated parents, live in a one-parent family and have more mother-reported problems compared to the rest of the Generation R sample. However, it is not likely that this selection of participants altered our results [40]. In ALSPAC, a longitudinal study comparable to Generation R, drop-out led to an underestimation of problem prevalence, but the association between determinants and outcome were not altered [41]. The drop-out did reduce the statistical power in the ALSPAC study, but we limited this effect by applying multiple imputation.

### Future research and implications

Based on our study, we have some recommendations for future research. As we focussed on psychosocial care in general, we recommend focussing more on the specific types and duration of psychosocial care in future research. Associations might be different for preventive, basic/community and specialised care, as demonstrated in comparable studies on children’s psychosocial care use [42, 43]. Furthermore, we recommend studying the predictive value of teacher-reported problems at multiple points in time. Problems reported closest to the care use show the strongest association, but problems earlier in life can contribute to early intervention strategies. Moreover, we recommend further research into the pathways teachers can support or provide for non-Western children towards psychosocial care. Pathways might be influenced by teacher characteristics such as the age, gender and ethnic background. Also awareness of available care, parenting support and services, personal experiences with mental health care and the attitude towards care might influence this pathway. Therefore, it is important to take these characteristics into account when exploring the pathways in future studies. Insight might reveal important further directions on how to improve access to psychosocial care for children with a non-Western background.

Our findings suggest that children who use care at 9 years old have relatively more problems at 6 years old, according to the teacher. Prevention strategies should focus on the teacher’s unique position, especially since the association is independent of mother-reported problems. In fact, teachers might have an important role in the identification and access to care of children with a non-Western background [25], particularly given that their parents are less likely to report problems and refer their child to psychosocial care [7, 8, 18].

## Conclusion

Our findings indicate that teachers might have an important role, next to parents, in the identification of emotional and behavioural problems and children’s access to psychosocial care. This may be particularly important for non-Western children, as they use less care than Western children, despite other research showing that they generally display higher levels of problems. Further research is warranted to improve our understanding of psychosocial care use by children with a Western and non-Western background.

## Supplementary Information

Below is the link to the electronic supplementary material.

Supplementary file1 (DOCX 18 KB)

## Data Availability

See the Generation R design paper [21]. Data is available when collaboration is established.
