# Supplementary material for: Teacher-reported emotional and behavioural problems and ethnic background associated with children’s psychosocial care use: a longitudinal population-based study
Source: Eur Child Adolesc Psychiatry. 2022 Jan 10;32(7):1263–71. doi: 10.1007/s00787-021-01937-w (PMC10276114; doi:10.1007/s00787-021-01937-w)
Supplement: Supplementary file 1 — Supplementary file1 (DOCX 18 KB) [file 787_2021_1937_MOESM1_ESM.docx]

Table SI – Testing for any moderation in the association between teacher-reported problems and psychosocial care use at nine years old (N=3,084)

| **Interaction term** | **P-value interaction term** |
| --- | --- |
| TRF­­_Total_ *Ethnic background | .899 |
| TRF­­_Externalising_ *Ethnic background | .599 |
| TRF_­­Internalising_ *Ethnic background | .644 |
| CBCL_Total_*Ethnic background | .123 |
| TRF­­_Total_*CBCL_Total_ | .172 |
| TRF­­_Externalising_*CBCL_Total_ | .359 |
| TRF_­­Internalising_*CBCL_Total_ | .533 |
| TRF*CBCL  CBCL*ethnic background  TRF*ethnic background  TRF­­_Total_*CBCL_Total_*ethnic background | .864  .455  .771  .561 |
| TRF*CBCL  CBCL*ethnic background  TRF*ethnic background  TRF­­_Externalising_*CBCL_Total_* ethnic background | .797  .540  .438  .586 |
| TRF*CBCL  CBCL*ethnic background  TRF*ethnic background  TRF_­­Internalising_*CBCL_Total_* ethnic background | .849  .217  .652  .987 |

Bold: represents p≤0.05. TRF: Teacher report form. CBCL: Child Behavior Checklist, representing mother-reported problems. Moderation tested in Model 3: Ethnic background, teacher-reported problems, age at visit research centre (outcome), sex, educational level mother and father, family situation, and mother-reported problems. All problem-related variables are dichotomous, representing children without emotional and behavioural problems and children with emotional and behavioural problems above the 83^rd^ percentile.

Table SII - Complete case analyses: Multivariable hierarchical logistic regression with presence of teacher-reported total emotional and behavioural problems, ethnic background and psychosocial care use at nine years old (N=2,388)

| *Overall problem level* | Univariate analyses  *OR (95% CI)* | Model 1  *OR (95% CI)* | Model 2  *OR (95% CI)* | Model 3  *OR (95% CI)* |
| --- | --- | --- | --- | --- |
| Teacher-reported problems  Presence of total problems^a^ | **3.79 (2.74-5.24)** | **4.07 (2.92-5.65)** | **3.86 (2.75-5.41)** | **3.52 (2.50-4.96)** |
| Ethnic background  Western (ref.)  Non-Western | 1.00  .76 (.54-1.08) | 1.00  **.63 (.44-.90)** | 1.00  **.60 (.41-.88)** | 1.00  **.58 (.40-.85)** |
| Mother-reported total problems | **2.61 (1.88-3.62)** | - | - | **2.23 (1.57-3.16)** |
| *Externalising problems* | Univariate analyses | Model 1 | Model 2 | Model 3 |
| TRF  Presence of externalising problems^a^ | **4.26 (3.09-5.87)** | **4.43 (3.21-6.12)** | **4.19 (3.01-5.84)** | **3.84 (2.74-5.38)** |
| Ethnic background  Western (ref.)  Non-Western | 1.00  .76 (.54-1.08) | 1.00  **.67 (.47-.96)** | 1.00  **.65 (.44-.94)** | 1.00  **.63 (.43-.92)** |
| Mother-reported total problems | **2.61 (1.88-3.62)** | - | - | **2.20 (1.55-3.12)** |
| *Internalising problems* | Univariate analyses | Model 1 | Model 2 | Model 3 |
| TRF  Presence of internalising problems^a^ | **2.20 (1.58-3.07)** | **2.26 (1.62-3.16)** | **2.21 (1.57-3.10)** | **2.04 (1.45-2.88)** |
| Ethnic background  Western (ref.)  Non-Western | 1.00  .76 (.54-1.08) | 1.00  .72 (.51-1.02) | 1.00  **.66 (.46-.97)** | 1.00  **.63 (.43-.92)** |
| Mother-reported total problems | **2.61 (1.88-3.62)** | - | - | **2.41 (1.71-3.39)** |

Bold: represents p≤0.05. OR=Odds Ratio for psychosocial care use at age nine years. 95% CI=95% confidence interval. TRF: Teacher report form. ^a^Teacher-reported total problems above the 83^rd^ percentile. Model 1: Ethnic background, TRF total problem score. Model 2: Model 1 + age at visit research centre (outcome), sex, educational level mother and father, family situation. Model 3: Model 2 + mother-reported emotional and behavioural problems.
